# Gender and Ethnicity Based Differences in Clinical and Laboratory Features of Myasthenia Gravis

**DOI:** 10.1155/2015/197893

**Published:** 2015-06-14

**Authors:** Fawzi Abukhalil, Bijal Mehta, Erin Saito, Sejal Mehta, Aaron McMurtray

**Affiliations:** ^1^Neurology Department, Los Angeles Biomedical Research Institute, 1124 West Carson Street, Building E5, Torrance, CA 90502, USA; ^2^Department of Neurology, Harbor-UCLA Medical Center, 1000 West Carson Street, Torrance, CA 90509, USA

## Abstract

*Background*. Previous reports describe ethnicity based differences in clinical and laboratory features between Caucasians and African Americans with myasthenia gravis. However, it is not known whether these findings apply to other ethnicities. *Methods*. Retrospective analysis of all patients treated for myasthenia gravis during a three-year period at a community based medical center. *Results*. A total of 44 patients were included, including 19 of Hispanic, 16 of African American, 6 of Caucasian, and 3 of Asian ethnicities. Female gender was more common among those with Hispanic, Asian, and African American ethnicities compared to Caucasian ethnicity (*p* = 0.029). Anti-acetylcholine receptor antibody subtypes demonstrated no significant ethnicity based differences in either generalized or ocular myasthenia gravis. A trend was noted towards greater frequency of blocking antibodies among Hispanics (52.6%) compared to African American (37.5%) and Caucasian (33.3%) patients (*p* = 0.059). Generalized but not ocular myasthenia patients showed greater frequency of anti-muscle specific kinase antibodies in Asians and Hispanics compared to African Americans and Caucasians (*p* = 0.041). *Conclusions*. The results of this study support the existence of ethnicity based differences in clinical and laboratory features of myasthenia gravis. Further study of genetic factors influencing clinical features of myasthenia gravis is indicated.

## 1. Introduction

Myasthenia gravis (MG) is the most common autoimmune disorder affecting the neuromuscular junction and is caused by production of autoantibodies that target postsynaptic acetylcholine receptors or associated protein kinases [1, 2]. One striking and well known clinical feature is the complex relationship between gender and onset age, with increased prevalence found in women younger than the third decade of life and men older than the sixth decade of life, with an overall female-to-male ratio of 3 : 2 [3, 4]. While the underlying etiology for gender based differences in onset age is not yet determined, it is likely that this finding relates to yet unidentified gender based genetic differences in regulation of immune system functioning, which serves to highlight the potential role that yet undetermined genetic factors may play in the clinical features of this disease.

In this study, we used ethnicity as a surrogate marker for the existence of possible underlying genetic differences influencing clinical characteristics of MG, with differences in clinical and laboratory features of MG between ethnic groups taken as evidence for the existence of underlying genetic factors that influence or control expression of clinical features. For example, previous studies have shown that the incidence of anti-acetylcholine receptor- (AChR-) negative MG, including those with muscle specific kinase (MuSK) positive antibodies, varies considerably according to ethnic background, with a reported frequency in Norwegian populations near 0% but as high as 49% in Turkey [5, 6]. Another recent study also reported the existence of ethnicity based differences between Caucasians and African Americans with MG in the United States and identified ethnicity based differences in the rates of anti-AChR antibody positivity, anti-MuSK positivity, frequency of abnormalities on repetitive nerve stimulation results, and differences in rates of clinical severity, as well as gender based differences in age of onset [7].

We hypothesized that since the proposed etiology for ethnicity based differences in clinical and laboratory features of MG between Caucasians and African Americans is underlying genetic differences [7], we would be able to detect similar ethnicity based differences in clinical and laboratory features among additional ethnicity groups as well. In particular, we were interested in ethnicity based differences affecting Hispanic patients, as this group represents one of the fastest growing segments of the population in the United States and has displayed an increasing prevalence of MG in recent years [8]. Identification of ethnicity based differences in clinical and laboratory features in MG is important since the genetic background differences inherently present between ethnicity based groups allow ethnicity to serve as a surrogate marker for the existence of genetic differences that underlie or control phenotypic expression. Identification of underlying genetic factors may eventually lead to new or improved treatments for patients of all ethnicities suffering from MG.

## 2. Materials and Methods

### 2.1. Participant Characteristics

This is a retrospective cross-sectional study of all adults diagnosed with or treated for MG in a community based neurology clinic during a three-year period between January 1, 2010, and January 1, 2013. Demographic data including ethnicity, age, gender, presence of comorbid medical conditions, presence of thymoma, and tobacco, alcohol, and illicit substance use was collected and analyzed through retrospective chart review. Institutional Review Board approval was obtained.

### 2.2. Diagnosis of Myasthenia Gravis

Diagnoses of generalized and ocular MG were determined by ICD-9 code and retrospective chart review. In brief, all patients met standard clinical diagnostic criteria for MG as determined by presence of one or more of the following characteristic symptoms or signs: diplopia, ptosis, dysarthria, difficulty in chewing, difficulty in swallowing, muscle weakness with preserved deep tendon reflexes or increased weakness during exercise, and dramatic improvement in signs and symptoms following administration of an anticholinesterase medication [9]. In addition to one of the above characteristic findings, all participants displayed positive results on at least one of the following serum based laboratory tests: anti-AChR antibodies (blocking, binding, or modulating) or anti-MuSK antibodies [10].

### 2.3. Statistical Analysis

Categorical demographic, clinical, and laboratory features were compared between groups using Pearson's Chi-Square tests or Fisher's exact test as appropriate. Continuous demographic, clinical, and laboratory features were compared between groups using analysis of variance (ANOVA). *p* values less than 0.05 were considered statistically significant and no correction for multiple comparisons was performed.

## 3. Results

A total of forty-four patients were included in the study, including 19 (43.2%) with Hispanic, 16 (36.4%) with African American, 6 (13.6%) with Caucasian, and 3 (6.8%) with Asian ethnicities. Overall, there were more women than men and the age of the participants ranged from 20 to 82 years but did not differ significantly between ethnicity groups (see Table 1). Gender ratios differed significantly by ethnicity, with the greatest percentage of females being among the Asian group (3/3, 100%), compared to 16 of 19 (84%) Hispanic participants, 8 of 16 (50%) African American participants, and 2 of 6 (33.3%) Caucasian participants (*p* = 0.029; see Table 1). No significant differences were detected between ethnicity groups for presence of comorbid medical conditions, psychiatric symptoms, prevalence of thymoma, or tobacco, alcohol, or illicit substance use (see Table 1). Disease severity was similar between ethnicity groups, with the majority of the patients having Myasthenia Gravis Foundation of America (MGFA) scale classification scores of 2a and 3a, and there were no MGFA class 4 or class 5 patients (see Table 1). Additionally, there were a total of three MGFA class 1 patients identified among the MuSK positive antibody patients (see Table 1).

Overall, an anti-AChR antibody test was positive in 63.64% of all MG patients and was positive more often among those with ocular MG (87.50%) compared to those with generalized MG (58.33%, *p* = 0.049). The anti-AChR antibody most likely to be positive was the binding type, which was positive in 59.09% of all patients and accounted for 41.94% of all anti-AChR antibodies identified (see Table 2). The AChR antibody that was least likely to be positive was the modulating type, which was present in 36.36% of all patients and accounted for 25.81% of all positive anti-AChR antibodies detected (see Table 2). While no significant ethnicity based differences were detected for anti-AChR subtypes among MG patients, there was a trend (*p* = 0.059) towards greater frequency of blocking antibodies among Hispanic patients (52.6%) compared to African American (37.5%) and Caucasian (33.3%) patients, and it is notable that the three Asian participants displayed only anti-MuSK antibodies and no anti-AChR antibodies (see Table 2). Overall, among the 18 patients who were tested for the presence of anti-MuSK antibodies, 5 (27.78%) tested positive, including 2 (10.52%) of those with Hispanic ethnicity and 3 (100%) with Asian ethnicity, compared to none (0.0%) in both African American and Caucasian groups (*p* = 0.041; see Table 2).

Ocular MG was present in 8 (18.18%) of the participants, and frequency of ocular MG did not differ significantly among the different ethnicity groups. However, none of the Asian participants were diagnosed with ocular MG (see Table 2). Also, no significant ethnicity based differences were detected for rates of positive anti-AChR antibodies or positive anti-MuSK antibodies for those with ocular MG (see Table 2).

## 4. Discussion 

In this study, we confirmed the existence of ethnicity based differences in gender distribution and serum antibody distribution among MG patients. These results support the presence of underlying genetic differences that affect aspects of phenotypic expression in MG, such as anti-AChR antibody type. It is possible that other aspects of the disease may show ethnicity based differences that were not detected in this study due to the small sample size and limited availability of data from patients of several ethnicities. A larger population based study, including data from a more diverse sample of participants, may help to elucidate additional clinical features that are similarly affected by ethnicity or demonstrate further evidence of genetic influences on phenotypic expression in MG.

Previous studies have reported the existence of ethnicity based differences in clinical and laboratory features of MG [5, 7]. A recent report by Oh et al. [7] described racial differences in clinical and laboratory features of MG between Caucasians and African Americans [7]. As discussed by Oh et al. [7], anti-AChR antibodies were more frequently positive in Caucasians (71%) compared to African Americans (59%). This is in agreement with the results from our sample, where anti-AChR antibodies demonstrated nonsignificant ethnicity related differences and were positive more frequently among Hispanics and Caucasians compared to African Americans and Asians. Their study also reported greater frequency of ocular myasthenia and positive repetitive nerve stimulation testing among African Americans compared to Caucasians, as well as differences in gender and age distributions between Caucasians and African Americans, with Caucasians having greater frequency of male gender and later age of onset [7]. In this study, we confirmed the greater frequency of male gender among Caucasians, but we did not have reliable age of onset information to either confirm or dispute this finding and we did not have information about repetitive nerve stimulation results. Additionally, while we did not detect any statistically significant differences in frequency of ocular MG between ethnicity groups, this may have been due to the small sample size of our study, and while not statistically significant, ocular MG occurred more frequently among those with Hispanic and African American ethnicities compared to Caucasians and Asians.

Anti-MuSK antibodies are also reported to vary between ethnic groups in MG. As discussed by Deymeer et al. [5], they found an overall rate of 49% for MuSK positive antibodies among MG patients, which is greater than the 27.78% rate of MuSK positive antibodies among the patients in this study. Previous reports of ethnicity based variation in MuSK antibodies showed greater frequency of positivity among African Americans (14%) compared to Caucasians (4%); and among patients with seronegative generalized MG the rates were 50% in African Americans and 16.7% in Caucasians [7]. The lower rate of MuSK positivity among Caucasians described in their study [7] also compares well to the rates identified in our study, in which 23.5% of patients overall were found to be MuSK positive, and although the differences were not statistically significant, the rates of MuSK positivity were greater among Hispanic and Asian participants than the Caucasian participants. However, we did not find a greater rate of MuSK positivity among African Americans compared to Caucasians, possibly related to the small sample size of our study.

This study has several limitations. First, the data was obtained exclusively from participants who visited an outpatient clinic and we were not able to obtain data from inpatients who may display more active disease. Consequently, this may have underestimated the true anti-AChR antibody positivity rates. Additionally, antibody positivity rates may have been underestimated due to the cross-sectional study design, since some patients that are initially anti-AChR antibody negative may seroconvert over time. However, these factors would be expected to have affected all ethnicity groups equally, so this is unlikely to have affected the overall results of the study and we feel confident that the ethnicity based differences identified in this study are indeed accurate. Another limitation of this study is the relatively small sample size with limited representation of some ethnicity groups which may have prevented detection of additional ethnicity based differences. Furthermore, clinical features were documented based on self-report and chart review, which also may have contributed to underreporting, especially regarding presence of comorbid psychiatric symptoms and use of tobacco, alcohol, or illicit substances. However, this type of underreporting error would also be expected to have affected all ethnicity groups equally and consequently also would not likely adversely affect the results of the study.

## 5. Conclusions

The results of this study confirm the existence of ethnicity based differences in clinical and laboratory features of MG. These results suggest a need for further study of genetic and biological factors involved in the pathogenesis of the disease, clinical features, disease course, and response to treatment. Identification of genetic factors underlying ethnicity based differences in clinical and laboratory features of MG may lead to development of new or improved treatments for this disorder and benefit patients of all ethnicities suffering from MG.

## Figures and Tables

**Table 1:** Ethnicity and clinical features in myasthenia gravis.

	Hispanic	African American	Caucasian	Asian	Sig.
Number (%)	19 (43.2%)	16 (36.4%)	6 (13.6%)	3 (6.8%)	N/A
Mean age in years (S.D.)	42.05 (16.02)	48.31 (17.51)	49.67 (11.48)	44.67 (6.51)	*p* = 0.610
Female gender	16 (84.2%)	8 (50.0%)	2 (33.3%)	3 (100%)	**p** = 0.029
Hypertension	7 (36.8%)	6 (37.5%)	3 (50.0%)	0 (0.0%)	*p* = 0.822
Diabetes	2 (10.5%)	2 (12.50%)	0 (0.0%)	0 (0.0%)	*p* = 0.862
Tobacco	0 (0.0%)	2 (12.50%)	0 (0.0%)	0 (0.0%)	*p* = 0.585
Alcohol	1 (5.3%)	3 (18.8%)	1 (16.7%)	0 (0.0%)	*p* = 0.658
Illicit substance	0 (0.0%)	0 (0.0%)	0 (0.0%)	0 (0.0%)	*p* = 0.279
Depression	1 (5.3%)	2 (12.50%)	1 (16.7%)	0 (0.0%)	*p* = 0.602
Anxiety	0 (0.0%)	1 (6.3%)	1 (16.7%)	0 (0.0%)	*p* = 0.218
Thymoma present	6 (31.6%)	2 (12.50%)	2 (33.3%)	0 (0.0%)	*p* = 0.381
Myasthenia Gravis Foundation of America scale score					
1	1 (5.3%)	0 (0.0%)	0 (0.0%)	2 (66.6%)	*p* = 0.107
2a	12 (63.2%)	12 (75.0%)	4 (66.6%)	1 (33.3%)	*p* = 0.247
2b	0 (0.0%)	0 (0.0%)	0 (0.0%)	0 (0.0%)	*p* = 0.279
3a	6 (31.6%)	4 (25.0%)	2 (33.3%)	0 (0.0%)	*p* = 0.463
3b	0 (0.0%)	0 (0.0%)	0 (0.0%)	0 (0.0%)	*p* = 0.279
4	0 (0.0%)	0 (0.0%)	0 (0.0%)	0 (0.0%)	*p* = 0.279
5	0 (0.0%)	0 (0.0%)	0 (0.0%)	0 (0.0%)	*p* = 0.279

**Table 2:** Ethnicity and laboratory features in myasthenia gravis.

	Hispanic	African American	Caucasian	Asian	Sig.
All myasthenia gravis					
Anti-AChR antibodies positive (%)					
Blocking	10 (52.6%)	6 (37.5%)	2 (33.3%)	0 (0.0%)	**p** = 0.059
Binding	13 (68.4%)	9 (56.3%)	3 (50.0%)	0 (0.0%)	*p* = 0.622
Modulating	7 (36.8%)	5 (31.3%)	3 (50.0%)	0 (0.0%)	*p* = 0.877
Anti-MUSK antibodies positive (%)	2 (10.5%)	0 (0.0%)	0 (0.0%)	3 (100%)	**p** = 0.017

Generalized myasthenia gravis					
Anti-AChR antibodies positive (%)					
Blocking	7 (46.67%)	5 (38.5%)	2 (40.0%)	0 (0.0%)	*p* = 0.514
Binding	9 (60.0%)	7 (53.9%)	3 (60.0%)	0 (0.0%)	*p* = 0.363
Modulating	4 (26.7%)	4 (30.8%)	2 (40.0%)	0 (0.0%)	*p* = 0.391
MUSK antibodies positive (%)	2 (13.3%)	0 (0.0%)	0 (0.0%)	3 (100%)	**p** = 0.041

Ocular myasthenia gravis					
Anti-AChR antibodies positive (%)					
Blocking	3 (75.0%)	1 (33.3%)	1 (100%)	n/a	*p* = 0.376
Binding	4 (100%)	2 (66.7%)	0 (0.0%)	n/a	*p* = 0.108
Modulating	3 (75.0%)	1 (33.3%)	1 (100%)	n/a	*p* = 0.376
Anti-MUSK antibodies positive (%)	0 (0.0%)	0 (0.0%)	0 (0.0%)	n/a	n/a
