# Corrigendum to “Gender and Ethnicity Based Differences in Clinical and Laboratory Features of Myasthenia Gravis”

**DOI:** 10.1155/2021/9862946

**Published:** 2021-05-29

**Authors:** Fawzi Abukhalil, Ayyaz Alam Sultan, Bijal Mehta, Erin Saito, Sejal Mehta, Aaron McMurtray

**Affiliations:** ^1^Neurology Department, Los Angeles Biomedical Research Institute, 1124 West Carson Street, Building E5, Torrance, CA 90502, USA; ^2^Dow University of Health Sciences, Dow Medical College, Karachi, Pakistan; ^3^Department of Neurology, Harbor-UCLA Medical Center, 1000 West Carson Street, Torrance, CA 90509, USA

In the article titled “Gender and Ethnicity Based Differences in Clinical and Laboratory Features of Myasthenia Gravis” [1], Ayyaz Alam Sultan was missing from the authors' list in error. With the agreement of all authors, Ayyaz Alam Sultan is added to the authors' list due to the contribution to data interpretation and manuscript editing. The corrected authors' list is shown above.
